# Erythropoiesis-Stimulating Agents in the Management of Anemia in Chronic Kidney Disease or Cancer: A Historical Perspective

**DOI:** 10.3389/fphar.2018.01498

**Published:** 2019-01-09

**Authors:** Matti Aapro, Pere Gascón, Kashyap Patel, George M. Rodgers, Selwyn Fung, Luiz H. Arantes, Jay Wish

**Affiliations:** ^1^Genolier Cancer Centre, Clinique de Genolier, Genolier, Switzerland; ^2^Division of Medical Oncology, Hospital Clinic, University of Barcelona, Barcelona, Spain; ^3^Carolina Blood and Cancer Care, Rock Hill, SC, United States; ^4^Division of Hematology and Hematologic Malignancies, Department of Internal Medicine, University of Utah School of Medicine, Salt Lake City, UT, United States; ^5^Supportive Care Biosimilars, Pfizer Inc., New York, NY, United States; ^6^Division of Nephrology, Indiana University Health, Indianapolis, IN, United States

**Keywords:** anemia, erythropoiesis-stimulating agents, cancer, kidney disease, biosimilars

## Abstract

Anemia is common in patients with cancer or with chronic kidney disease (CKD). Although the introduction of erythropoiesis-stimulating agents (ESAs) has transformed the management of anemia, their use has been complicated by a number of factors including frequent guideline updates, safety concerns and, in the United States, a Risk Evaluation and Mitigation Strategy (REMS) program, which aimed to ensure that the benefits of ESAs outweigh the risks. Many previous concerns around ESA use in cancer and CKD have been addressed by the reassuring results of post-approval studies, and biosimilar ESAs have been used in Europe for many years, with safety and efficacy profiles similar to originator products. This review describes the evolution of the use of ESAs from approval to the present day, discussing results from clinical studies of ESAs in cancer and CKD, and the influence of these findings on product labeling and guideline updates. We also discuss the impact of the introduction of ESA biosimilars in Europe, bringing cost savings and increased access to patients.

## Introduction

Over 100 years ago, Carnot and Deflandre speculated on the existence of a humoral factor produced in response to anemia. Rabbits infused with serum from anemic animals showed an increase in red blood cells, leading to the conclusion that erythropoiesis is regulated by a blood-borne factor (Bunn, 2013). This finding was later confirmed by Erslev (1953) and further studies demonstrated the kidneys to be the main source of production of this factor, now known as erythropoietin (Jacobson et al., 1957; Nathan et al., 1964). Following identification of the erythropoietin gene, ESAs were soon being mass-produced, reducing anemia in the same manner as the human protein erythropoietin, i.e., by stimulating the proliferation and differentiation of red blood cell progenitors (burst-forming unit-erythroids and colony-forming unit-erythroids), and preventing apoptosis (Elliott et al., 2008; Lodish et al., 2010).

Since the discovery of erythropoietin, ESAs have been used to treat anemia in various patient groups, including patients with cancer or CKD, where anemia is a common problem. For example, the European Cancer Anemia Survey, published in 2004, reported a 39.3% prevalence of anemia in 15,367 cancer patients at enrollment (Ludwig et al., 2004) and a recent paper reported the presence of anemia in 3,962 (89.5%) of 4,426 patients during the course of receiving chemotherapy for solid tumors (Xu et al., 2016). In non-Hodgkin lymphoma or Hodgkin lymphoma, 30–40% of patients are anemic at diagnosis, while up to 70% of patients with multiple myeloma are anemic at diagnosis, with higher rates in myelodysplastic syndromes (Bennett et al., 2010). An analysis conducted in 2014 in the United States found anemia to be twice as prevalent in people with CKD (15.4%) than in the general population (Stauffer and Fan, 2014). Anemia prevalence increased with stage of kidney disease (8.4% at stage 1 through to 53.4% at stage 5) (Stauffer and Fan, 2014).

While homologous blood transfusion might be a quick way to alleviate the symptoms of anemia, it is associated with a number of hazards, including the transmission of infection and iron overload (Porter, 2001; Jabbour et al., 2008). Since their introduction to the market in the late 1980s, ESAs have revolutionized the management of anemia, with improvements in QoL and mortality associated with the correction of anemia of CKD patients (Cella and Bron, 1999; Ma et al., 1999). Here, we review the history of ESA use in patients with cancer or CKD, discussing changing guidance, benefits, limitations and appropriate use of ESAs in these patient groups. See Table 1 for a chronological listing of the key guidelines consulted.

**Table 1:** Guidelines and recommendations reviewed (1997–2018).

Guideline	Year	Key findings/Recommendations for ESA use
*Oncology:*		
ASCO/ASH	2002	Initiate when Hb < 10 g/dL and in less severe anemia (10–12 g/dL) determined by clinical circumstances. Target no higher than 12 g/dL
NCCN	2002	Target Hb of 12 g/dL
EORTC	2004	Slight elevation in the risk of thromboembolic events and hypertension with ESA use
FDA	2004	Target Hb levels no higher than 12 g/dL
EORTC	2006	Initiate at Hb levels of 9–11 g/dL, target Hb level 12–13 g/dL
FDA	2007	Black box warning to avoid Hb levels greater than 12 g/dL
NCD	2007	Limit ESA use in non-renal disease indications
ASCO/ASH	2007	Initiate when Hb < 10 g/dL, and in less severe anemia (Hb > 10 g/dL and <12 g/dL), use determined by clinical circumstances; cautioned against ESA use in cancer patients not receiving chemotherapy
FDA	2008	Initiate when Hb is ≥10 g/dL. Removed upper limit of 12 g/dL; patients treated with chemotherapy with curative intent excluded from ESA treatment
ASCO/ASH	2010	Lowest dose to achieve an increase in Hb to the lowest level for transfusion avoidance. Recommended ESAs as an option in CIA and Hb < 10 g/dL. Caution advised when used with chemotherapy in diseases associated with increased risk of thromboembolic complications
ESMO	2010	Hb limit of 12 g/dL; ESAs should be carefully reconsidered in patients with a high risk of thromboembolic events, used with caution in liver disease, and not given to patients with ESA hypersensitivity or poorly controlled hypertension
NCCN	2018	Maintain lowest Hb level to avoid transfusion. Avoid increases >1 g/dL in any 2-week period. Removed need to consult REMS
ESMO	2018	Target Hb level of 12 g/dL, initiated at <10 g/dL (symptomatic anemia) or <8 g/dL (asymptomatic anemia). Iron therapy should be given before and/or during ESA therapy in the case of absolute or functional iron deficiency. No clinical evidence for an effect of ESAs on stimulating disease progression or relapse when used within label in cancer patients
*Nephrology:*		
NKF-DOQI	1997	Target Hb level of 11–12 g/dL
FDA	2007	Black box warning recommending maintenance of Hb levels within the range of 10–12 g/dL for anemic patients with CKD
ERBP	2010	Target Hb level of 11–12 g/dL in CKD patients, do not intentionally exceed 13 g/dL
FDA	2011	Removed target Hb range of 10–12 g/dL; recommended use of the lowest ESA dose to reduce the need for transfusions
KDIGO	2012	For CKD patients with Hb concentration ≥ 10.0 g/dL, ESA therapy should not be initiated. Upper target limit of 11.5 g/dL. Individualization of therapy will be necessary because some patients may have improvements in QoL at Hb concentrations above 11.5 g/dL and will be prepared to accept the risks
NICE	2015	Target Hb range of 10–12 g/dL
Renal Association	2017	Target Hb range of 10–12 g/dL


*ASCO, American Society of Clinical Oncology; ASH, American Society of Hematology; ERBP, European Renal Best Practice; ESMO, European Society for Medical Oncology; FDA, United States Food and Drug Administration; KDIGO, Kidney Disease Improving Global Outcomes; NCCN, National Comprehensive Cancer Network; NCD, National Coverage Determination; NICE, National Institute for Health and Care Excellence; NKF-DOQI, National Kidney Foundation Dialysis Outcomes Quality Initiative; REMS, Risk Evaluation and Mitigation Strategy.*

## Clinical History of ESA Use in Oncology, Limitations and Guidelines

In 2002, ASCO and ASH published evidence-based guidelines on the use of epoetin in cancer (Rizzo et al., 2002). These guidelines recommended use in patients with chemotherapy-associated anemia with Hb concentrations below 10 g/dL, and in less severe anemia (Hb 10–12 g/dL) determined by clinical circumstances. It was suggested that Hb levels can be elevated to (or near) a concentration of 12 g/dL, with titration of ESAs to maintain that level, or recommencement of dosing when the Hb level falls to near 10 g/dL. The guidelines also stated that insufficient evidence to date support the “normalization” of Hb levels to above 12 g/dL. The 2002 NCCN guidelines on anemia recommended that Hb levels below 11 g/dL should prompt investigation of the symptoms of anemia, and symptomatic patients treated with ESAs or red blood cell transfusion (Rodgers, 2012). The guidelines recommended a target Hb concentration of around 12 g/dL and discussed the possible benefits to QoL associated with ESA use (Rodgers, 2012). In 2003, shortened survival with ESA treatment in patients with head and neck cancer was reported in the ENHANCE trial (Henke et al., 2003). In this trial of 351 patients, one group received epoetin beta in addition to radiotherapy, while the other group received placebo with radiotherapy. Eighty-two percent of patients receiving epoetin reached Hb levels higher than 14 g/dL (women) or 15 g/dL (men) compared with 15% of patients receiving placebo. Locoregional PFS was worse with epoetin compared with placebo, and the authors concluded that erythropoietin use in patients undergoing curative cancer treatment should be studied in carefully controlled trials (Henke et al., 2003). It is important to note that ESAs were used off-label, as no patient received chemotherapy.

The first Cochrane Database meta-analysis on ESA treatment outcomes in cancer patients (2004) reported a reduction in the need for blood transfusions with ESA use and a reduction in the number of units transfused (Bohlius et al., 2004). Also in 2004, the EORTC published guidelines for ESA use in anemic patients with cancer, which reported a slight elevation in the risk of thromboembolic events and hypertension in patients with CIA receiving ESAs (Bokemeyer et al., 2004). Based on emerging safety data, the FDA, in 2004, recommended target Hb levels no higher than 12 g/dL (Luksenberg et al., 2004). An early report on the BEST (Leyland-Jones and BEST Investigators and Study Group, 2003), showing decreased 12-month survival among patients with metastatic breast cancer with epoetin alfa treatment, was discussed in the 2005 NCCN guidelines (Rodgers, 2012), and the full results of the BEST study were published in 2005 (Leyland-Jones et al., 2005). The objective of this trial was to compare the effect of managing Hb levels between 12 and 14 g/dL with epoetin alfa vs. placebo (where Hb levels were not managed) in patients with metastatic breast cancer receiving first-line chemotherapy. Maintaining high Hb levels was associated with a significantly lower 12-month OS (Leyland-Jones et al., 2005).

The EORTC guidelines of 2006 reported no clear link between ESA use in conjunction with chemotherapy or radiotherapy on survival, local tumor control, time to progression, and PFS, although a slight elevation in the risk of thromboembolic events and hypertension in CIA with ESA treatment was again discussed (Bokemeyer et al., 2007). The guidelines recommended that, in cancer patients receiving chemotherapy and/or radiotherapy, ESAs should be initiated at Hb levels of 9–11 g/dL, and the target Hb concentration should be 12–13 g/dL.

Based on accumulating evidence, in 2007 the FDA issued a “black box” warning recommending avoidance of Hb levels greater than 12 g/dL, and the Centers for Medicare and Medicaid Services made an NCD to limit coverage of ESAs for non-renal disease indications, which was implemented in 2008 (Center for Medicare and Medicaid Services, 2007; US Food and Drug Administration, 2007b, 2017). The 2007 ASCO/ASH guidelines cautioned against ESA use in cancer patients not receiving chemotherapy due to reports of increased thromboembolic risks and decreased survival in such situations (Rizzo et al., 2008). ESAs were recommended as a treatment option in CIA when the Hb level approached or fell below 10 g/dL, and in less severe anemia (Hb > 10 g/dL and < 12 g/dL), with use determined by clinical circumstances. In myeloma, non-Hodgkin lymphoma, or chronic lymphocytic leukemia, the advice was to begin treatment with chemotherapy and/or corticosteroids and observe the hematologic outcomes achieved through tumor reduction before considering epoetin (Rizzo et al., 2008).

In 2008, the FDA lowered the Hb target at which to initiate ESA treatment to less than 10 g/dL and removed “…or exceeds 12 g/dL” from its guidance, and patients treated with chemotherapy with curative intent were excluded from ESA treatment (Hagerty, 2008). The next year, a Cochrane Database meta-analysis reported an increase in on-study mortality and worsened OS associated with ESAs (Bohlius et al., 2009). The 2010 ASCO/ASH guidelines advised that the lowest dose to achieve an increase in Hb to the lowest level required to avoid transfusions should be used (Rizzo et al., 2010). These guidelines recommended ESAs as an option for patients with CIA and Hb concentration lower than 10 g/dL, and advised caution when using with chemotherapeutic agents in diseases associated with increased risk of thromboembolic complications (Rizzo et al., 2010).

In 2010, ESMO released clinical practice guidelines for ESA use in treating anemia in cancer patients. The guidelines advised that Hb levels should not exceed 12 g/dL, and that ESAs should be carefully reconsidered in patients with a high risk of thromboembolic events, used with caution in liver disease, and not given to patients with ESA hypersensitivity or poorly controlled hypertension (Schrijvers et al., 2010). The guidelines concluded that the evidence of the influence of ESAs on tumor progression and OS in anemic cancer patients was unclear.

**FIGURE 1:**
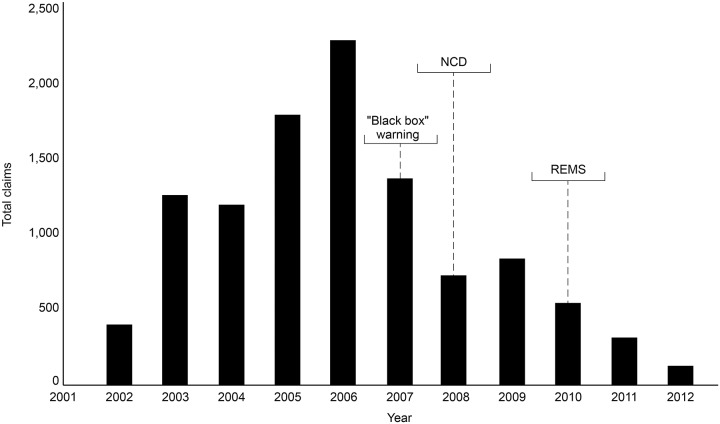
South Carolina Medicaid claim data (2002–2012). Reductions in the number of claims for ESAs were observed over the period 2006–2012, indicating the “black box” warning resulted in reductions in ESA use. ESA, erythropoiesis-stimulating agent; NCD, National Coverage Determination; REMS, Risk Evaluation and Mitigation Strategy.

**FIGURE 2:**
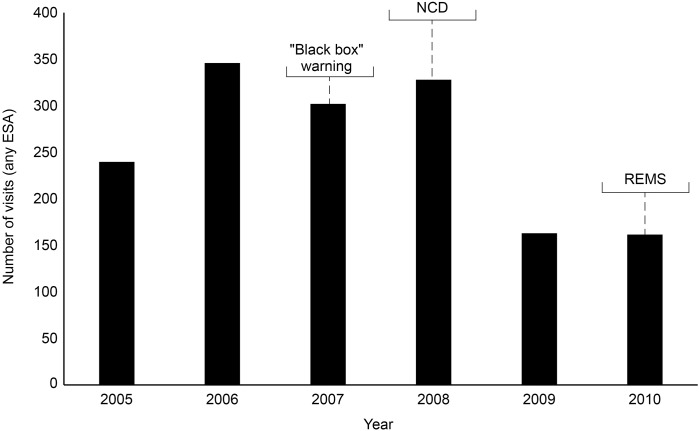
Electronic health records in Cerner Database: overall annual trend in the number of visits with ESA use per reporting hospital. The number of cases for which an ESA was prescribed increased 44% from 240 in 2005 to 346 in 2006. ESA use decreased 13% in 2007 from the previous year; then use increased by 9% to 328 cases in 2008. The largest reduction was seen in 2009 with a 50% reduction from 2008. Use remained low from then through 2010. ESA, erythropoiesis-stimulating agent; NCD, National Coverage Determination; REMS, Risk Evaluation and Mitigation Strategy.

The ESA REMS plan was implemented by the FDA in 2010. This was a process aimed at ensuring that the benefits outweigh the risks when prescribing ESAs, although REMS did not apply to CKD patients. Healthcare providers and hospitals were required to become certified in the program, provide counseling to each patient, and have patients complete a Patient and Healthcare Provider Acknowledgement Form before treatment. By 2011, following the “black box” warning, the NCD, and REMS, there had been a decline in the proportion of patients receiving chemotherapy using ESAs, an increase in the proportion of patients where ESAs were initiated at Hb levels <10 g/dL, and an increase in the proportion of patients receiving ESAs at dosages consistent with product labeling (US Food and Drug Administration, 2017). An analysis of total ESA claims from the merged South Carolina Medicaid Cancer Registry dataset for cancer patients on chemotherapy from 2002 to 2012 reported a decline in ESA claims from 2006 through 2012, showing reductions over the period encompassing the “black box” warning of 2007, the NCD, and the introduction of REMS (Figure 1) (Noxon et al., 2014). Claims peaked at 2,243 in 2006, but this figure had decreased to 714 by 2008. A monthly breakdown showed a peak of 250 claims in May 2006, decreasing to 7 by December 2012. Another in-depth report involved an analysis of electronic health records in the Cerner Database from January 1, 2005, to June 30, 2011, reporting the use of epoetin alfa and darbepoetin alfa in CKD and chronic anemia. Between these dates, there were 111,363 encounters of ESA use, representing 86,763 patients admitted to Cerner Health. Overall, ESA use in this sample decreased by 33% over the period studied, with the biggest reduction seen in 2009, following the NCD of 2008 (Figure 2) (Seetasith, 2013). At the time of writing, we were unable to source detailed relevant data post-2012 on these measures of ESA use.

Following implementation of the REMS program, a number of studies were published reporting no impact of ESAs on OS in cancer patients receiving ESAs for CIA (see Table 2 for a summary). Although the REMS strategy had been in place for a number of years, by 2017 the FDA determined it was no longer necessary as prescribers demonstrated acceptable knowledge of product risks, and drug utilization data indicated appropriate prescribing of ESAs (US Food and Drug Administration, 2017). The NCCN guidelines for 2018 reflected the lifting of the REMS strategy by removing the need to consult REMS when considering ESA use (National Comprehensive Cancer Network [NCCN], 2018).

**Table 2:** Recent studies reporting survival data for ESAs in CIA.

Source	Tumor type	Patients (*n*)	Results
Untch et al., 2011	Breast	733	No effect – DFS, OS
Nagel et al., 2011	SCLC	73	No effect – PFS, OS
Fujisaka et al., 2011	Lung/Gyn	186	No effect – OS
Moebus et al., 2013	Breast	1284	No effect – RFS, OS
Nitz et al., 2014	Breast	1234	No effect – EFS, OS
Mountzios et al., 2016	Solid tumors	630	No effect – PFS, OS
Leyland-Jones et al., 2016	Met breast	2098	No effect – OS; No effect – PFS (independent review)
Nagarkar et al., 2018	NSCLC	2516	No effect – PFS, OS


*CIA, chemotherapy-induced anemia; DFS, disease-free survival; EFS, event-free survival; ESAs, erythropoiesis-stimulating agents; Gyn, gynecological; Met, metastatic; NSCLC, non-small cell lung cancer; OS, overall survival; PFS, progression-free survival; RFS, relapse-free survival; SCLC, small cell lung cancer. Untch et al. (2011): randomized Phase 3 trial of chemotherapy in breast cancer with or without darbepoetin (target Hb 12.5–13 g/dL in darbepoetin-treated patients). Addition of darbepoetin resulted in no differences in 3-year disease-free survival and OS. Nagel et al. (2011): randomized Phase 2 study involving chemotherapy in limited or extensive SCLC with or without darbepoetin (target Hb 12–13 g/dL in darbepoetin-treated patients); no differences in PFS or 1-year survival rates reported between treatment arms. Fujisaka et al. (2011): randomized Phase 3 trial of epoetin beta in patients receiving chemotherapy for lung/gynecological cancer (target Hb under 12 g/dL); no difference in 1-year survival rates reported between ESA treated vs. untreated. Moebus et al. (2013): in a second randomization of an intense dose-dense chemotherapy arm, outcome in primary breast cancer with or without addition of epoetin alfa (target Hb 12.5–13 g/dL) was studied. Epoetin treatment had no impact on OS or RFS at median follow-up of 62 months. Nitz et al. (2014): randomized Phase 3 trial in patients with node-positive primary breast cancer receiving chemotherapy with or without darbepoetin alfa. Initially, Hb levels were maintained between 13 and 14 g/dL, but this was modified to 12 g/dL after the label amendment of 2008. At 39 months, darbepoetin treatment had no impact on either EFS or OS. Mountzios et al. (2016): randomized Phase 3 trial investigating administration of ESAs in patients with solid tumors and CIA with a median follow-up of 7 years. Patients in one group received prophylactic ESA with iron supplementation (target Hb 14 g/dL). If Hb levels exceeded 14 g/dL, ESAs were discontinued and resumed when levels were <12 g/dL. In the other group, patients received iron supplementation with ESAs in a Hb-based manner, initiated only if Hb levels were <11 g/dL during chemotherapy, stopping at 13 g/dL. No differences in DFS, PFS, or OS were reported between the two groups with respect to the presence or absence of anemia at entry to study, or the manner in which ESAs were given (prophylactic vs. Hb-based). Leyland-Jones et al. (2016): epoetin alfa in anemic patients receiving chemotherapy for metastatic breast cancer. Patients received either epoetin alfa or best standard of care alongside chemotherapy. ESA treatment had no effect on OS. Although investigator-determined PFS was reported to be decreased by ESAs, an independent review determined PFS was not decreased. Nagarkar et al. (2018): evaluated non-inferiority of darbepoetin alfa (DAR) versus placebo for OS and PFS in anemic patients with NSCLC treated to a 12 g/dL hemoglobin ceiling. Darbepoetin was non-inferior to placebo for OS and PFS and significantly reduced odds of transfusion or hemoglobin ≤ 8 g/dL in anemic patients with NSCLC receiving myelosuppressive chemotherapy.*

Recently, the 2018 ESMO Clinical Practice Guidelines for the management of anemia and iron deficiency in patients with cancer (Aapro et al., 2018a) reported no clinical evidence for an effect of ESAs on stimulating disease progression or relapse when used within label. For ESA therapy in patients with solid tumors and hematologic malignancies, the guidelines recommend a target Hb level of 12 g/dL, initiated at <10 g/dL (symptomatic anemia) or <8 g/dL (asymptomatic anemia). The guidelines also advise that iron therapy should be given before and/or during ESA therapy in the case of absolute or functional iron deficiency. Backed by evidence from more than 2,500 ESA-treated patients, the 2018 guidelines also highlight that epoetin alfa is now indicated by the EMA for the treatment of anemia in low- or intermediate-1-risk primary myelodysplastic syndrome patients. The similar safety and efficacy of EMA-approved originator ESA products and biosimilars is also mentioned.

## Clinical History of ESA Use in Nephrology, Limitations and Guidelines

The first ESA approved in Europe for dialysis and non-dialysis patients with CKD was Eprex^®^ (epoetin alfa, Ortho Biotech) in 1988, and the first ESA approved for the United States dialysis market was Epogen^®^ (epoetin alfa, Amgen, Thousand Oaks, CA, United States), in 1989. Epogen^®^ was licensed for “treatment of anemia associated with chronic renal failure, including patients on dialysis (end-stage renal disease) and patients not on dialysis.” Labeling was expanded in 1993 to include a supplemental indication for the treatment of anemia associated with cancer chemotherapy. The longer-acting ESA, Aranesp^®^ (darbepoetin alfa, Amgen), was licensed in the United States in 2001 (Luksenberg et al., 2004).

In the late 1990s/early 2000s, interesting events underscored the importance of manufacturing standards in the production of biologics, with unexpected reports of pure red cell aplasia primarily associated with subcutaneous Eprex^®^ in patients with CKD (Bennett et al., 2004), leading health authorities in Europe, in 2002, to contraindicate subcutaneous Eprex^®^ in patients with CKD (Bennett et al., 2004). Following intensive investigation, it was considered that a switch to a new stabilizing agent (polysorbate 80) in 1998 might have resulted in increased immunogenicity through interaction with leachates from uncoated rubber stoppers of prefilled syringes, and possible protein denaturation or aggregate formation resulting from the polysorbate 80 formulation being more susceptible to stress conditions (such as insufficient attention to the cold supply chain) (McKoy et al., 2008). The manufacturers replaced the rubber stoppers with Teflon^®^-coated stoppers and, following the adoption of processes for its appropriate handling, storage, and administration, the exposure-adjusted incidence of pure red cell aplasia decreased worldwide by 83% (Bennett et al., 2004). By 2006, the contraindication for subcutaneous administration of Eprex^®^ in CKD patients had been lifted (Macdougall et al., 2015).

Throughout the 1990s, clinical practice guidelines for ESAs were developed and evolved through a focus on evidence-based medicine. In 1990, the Cooperative Multicenter EPO Clinical Trial Group reported a Phase 3 trial including more than 300 dialysis patients (Evans et al., 1990). Treatment with epoetin alfa resulted in improvements in many QoL measures, with benefits beyond the avoidance of transfusion and iron overload, including improvements in energy, sleep, and well-being (Evans et al., 1990). In 1997, the workgroup for the NKF-DOQI clinical practice guidelines for treating anemia in chronic renal failure (National Kidney Foundation Dialysis Outcomes Quality Initiative [NKF-DOQI], 1997) recommended hematocrit levels of 33–36% corresponding to a target Hb level of 11–12 g/dL, which was higher than the target of 30–33% set by the FDA in 1989; in 1994, the FDA increased the target hematocrit level range to 30–36% (Ma et al., 1999).

A number of studies in the nephrology arena have been pivotal in further shaping guidelines. In a double-blind study, Parfrey et al. (2005) randomized 596 incident hemodialysis patients to two groups, targeting Hb levels of 13.5–14.5 g/dL or 9.5–11.5 g/dL (Parfrey et al., 2005). Although the group with higher Hb levels had a greater incidence of cerebrovascular events, this group showed an improved QoL outcome (in terms of vitality) (Parfrey et al., 2005). The Correction of Hemoglobin and Outcomes in Renal Insufficiency (CHOIR) study of 2006 randomized 1,432 CKD patients to target Hb levels of 13.5 g/dL vs. 11.3 g/dL with epoetin alfa treatment (Singh et al., 2006). At follow-up, composite cardiovascular endpoints (including mortality) were significantly increased in the high-level Hb group. Although improvements in QoL were seen on correction of anemia, there was no difference in QoL between the two groups. The authors concluded that targeting higher Hb levels was associated with increased risk, with no incremental improvement in QoL. In Europe, the CREATE study randomized 603 CKD patients to early therapy and a target Hb of 13–15 g/dL or to “salvage” therapy and a target of 10.5–11.5 g/dL, only after the Hb level had decreased to less than 10.5 g/dL (Drueke et al., 2006). There were no differences between groups in primary cardiovascular and survival outcomes, although patients in the high-level Hb group showed hastened progression to dialysis, more hypertensive episodes, and improved QoL scores (Drueke et al., 2006).

The results of CHOIR and CREATE prompted the FDA, in 2007, to direct manufacturers of ESA products in the United States to add a “black box” warning recommending the maintenance of Hb levels within the range of 10–12 g/dL for anemic patients with CKD (US Food and Drug Administration, 2007b). Also in 2007, the FDA performed an analysis of CHOIR and also of the Normal Hematocrit Trial, which was a study involving 1,233 hemodialysis patients with cardiac disease comparing the survival advantage of achieving and maintaining normal hematocrit levels (42%) vs. low levels (30%) with epoetin alfa (Besarab et al., 1998). This trial was stopped early as a result of the expectation that patients in the normal arm would not have better outcomes than those in the low-level arm. In its analysis of CHOIR and the Normal Hematocrit Trial, the FDA reported an inverse relationship between achieved Hb level and cardiovascular events, finding that ESAs increased adverse outcomes when Hb levels changed at a rate greater than 0.5 g/dL per week, leading the FDA to caution against a rise in Hb greater than 1 g/dL over a 2-week period (US Food and Drug Administration, 2007a). A secondary analysis of CHOIR actually found ESA dose to be the major predictor of AEs, when the data were adjusted for ESA dose and patients not achieving their Hb target (Szczech et al., 2008). Those patients achieving target Hb levels had better outcomes than those who did not, and no increased risk of higher Hb levels was observed (Szczech et al., 2008).

The TREAT trial randomized anemic patients with type 2 diabetes and CKD to darbepoetin alfa treatment, with a target Hb level of 13 g/dL (Group 1), or the administration of placebo (Group 2), with rescue darbepoetin alfa given to patients in Group 2 only if Hb levels dropped below 9 g/dL (Pfeffer et al., 2009). Although there was a significantly higher incidence of stroke in Group 1, cardiovascular events, death, and QoL scores were similar for both groups (Pfeffer et al., 2009). Shortly after the publication of TREAT, the Anemia Working Group of the ERBP recommended targeting Hb levels of 11–12 g/dL in CKD patients, and to not intentionally exceed 13 g/dL (Locatelli et al., 2010). In 2011, the FDA removed the target Hb range of 10–12 g/dL, and recommended that the lowest ESA dose to reduce the need for transfusions should be used (US Food and Drug Administration, 2011). The following year, the 2012 guidelines of the Kidney Disease Improving Global Outcomes (Kidney Disease Improving Global Outcomes [KDIGO], 2012) advised that for adult CKD patients with Hb concentration ≥10.0 g/dL, ESA therapy should not be initiated. For maintenance therapy an upper limit of 11.5 g/dL was suggested. In a non-graded recommendation, KDIGO suggested that individualization of therapy will be necessary because some patients may have improvements in QoL at Hb concentrations above 11.5 g/dL and will be prepared to accept the risks. More recently, the NICE guidelines (National Institute for Health and Care Excellence [NICE], 2015), and the Renal Association clinical practice guidelines on anemia of CKD (Mikhail et al., 2017), both suggested a Hb target range of 10–12 g/dL.

Despite changes in guidance, the question of whether focus should be directed on avoiding high Hb levels or avoiding high ESA doses in ESA-resistant patients remained. A meta-regression analysis published in 2013 examined the association of ESA dose with adverse outcomes in CKD, independent of the target or Hb level achieved (Koulouridis et al., 2013). In 12,956 patients, all-cause mortality was associated with higher total-study-period mean ESA dose and higher first-3-month mean ESA dose. Total-study-period mean ESA dose and first-3-month ESA dose remained significant after adjusting for target Hb or first-3-month mean Hb, respectively. Hypertension, stroke, and thrombotic events, including dialysis vascular access-related thrombotic events, were increased with higher total-study-period mean ESA dose (Koulouridis et al., 2013).

**FIGURE 3:**
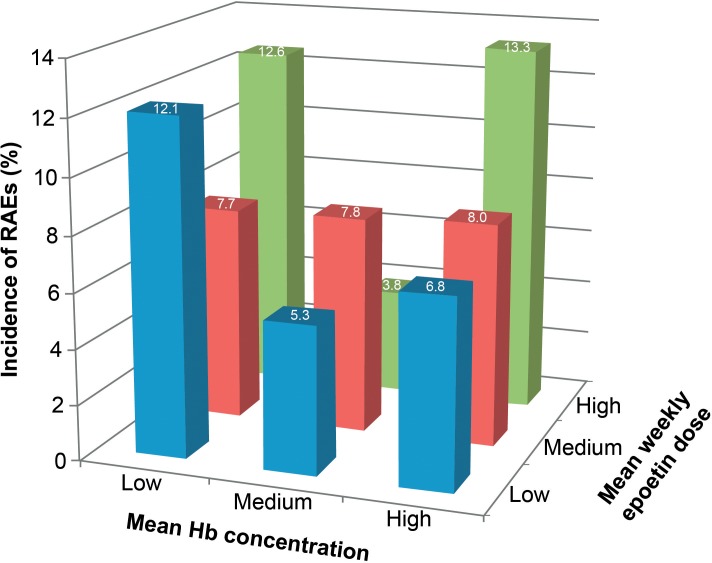
Relationship between reportable adverse events (RAEs) and the combined effects of hemoglobin (Hb) concentration and dose of epoetin theta therapy in patients with anemia associated with chronic kidney disease. RAEs were predefined cardiovascular events selected from the *Medical Dictionary for Regulatory Activities* classification system and included cardiac disorders, cardiac failure, myocardial infarction and ischemic stroke, and respective subterms. Hb, hemoglobin. Reprinted from Lammerich et al. (2016) with permission from Elsevier.

Data continue to be published in the nephrology literature regarding ESA dose vs. Hb concentration on patient outcomes. In 2016, data from a prospective, non-interventional, multinational cohort study of 1,039 consecutive patients with advanced or end-stage renal disease receiving epoetin theta was published (Lammerich et al., 2016). Data on Hb concentrations and reportable AEs (RAEs) were collected and the incidence of AEs examined *post hoc* according to tertiles for individual mean Hb concentration (≤10.7, >10.7–11.47, and >11.47 g/dL for low, intermediate, and high) and mean weekly epoetin theta dosage (≤62, >62–125, and >125 IU/kg/week for low, intermediate, and high). Intermediate Hb concentrations were associated with the lowest incidence of RAEs, and the incidence of ischemic stroke was 0.6% at both low and intermediate Hb concentrations, and 1.5% at high Hb concentrations. Patients in the high-dose/high-Hb group had the greatest risk for cardiovascular RAEs or ischemic stroke (13.3%), followed by high dose/low Hb (12.6%) and low dose/low Hb (12.1%). The risks for RAEs were lowest at low dose/intermediate Hb (5.3%) and high dose/intermediate Hb (3.8%) (Figure 3). The authors concluded that the lowest approved, effective dose of epoetin theta should be used (Lammerich et al., 2016).

## The Advent of Biosimilars

Although the appropriate risk-benefit ratio of ESAs has been established based on the current labeling and guidelines, access remains a significant problem in many areas of the world, with barriers including issues related to insurance coverage, drug availability, and cost (Lammers et al., 2014; Socinski et al., 2015). However, biosimilars have reduced development costs as a result of a less extensive trial program in comparison to originator products, bringing potential cost savings for patients and healthcare providers (Blackstone and Joseph, 2013; Singh and Bagnato, 2015).

An analysis in 2012 compared the cost efficiency of various originator epoetins vs. a biosimilar, Binocrit^®^ (epoetin alfa), across the EU G5 countries (France, Germany, Italy, the United Kingdom, and Spain) (Aapro et al., 2012). The cost of ESA treatment for 15 weeks was calculated for a single patient with cancer receiving chemotherapy; biosimilar epoetin alfa was calculated to cost €4,643 (30,000 IU) and €6,178 (40,000 IU) on average. Estimates for originator epoetin alfa treatment were €7,168 (40,000 IU), and for originator epoetin beta the cost was €7,389 (30,000 IU), with even higher costs for darbepoetin alfa. The savings from treating patients with biosimilar epoetin alfa (40,000 IU) were 13.8% over originator epoetin alfa, 16.4% over originator epoetin beta, and up to 33% over originator darbepoetin alfa (Aapro et al., 2012).

Biosimilar competition in the market not only brings lower prices but can also reduce the cost of originator products. An analysis of the European market, where various biosimilars have been available for >10 years, has shown increased treatment utilization and reduced treatment-day prices following the introduction of biosimilars in several therapy areas (Quintiles IMS, 2017). The analysis also shows that, in 2015 in the EU, biosimilar ESAs accounted for 62% of the market vs. reference product (Quintiles IMS, 2017). The cost savings associated with biosimilar use could be reallocated to enhance patient access to other therapies. For example, a simulation of the budgetary impact of ESA use in EU G5 countries involved a hypothetical panel of 100,000 patients, with three models estimating the number of patients who could be provided with rituximab, bevacizumab, or trastuzumab therapy from the cost savings of using biosimilar erythropoietin (Abraham et al., 2014). Under fixed dosing and assuming 100% conversion, the savings were over €110 million, translating to an additional ∼17,000 treatments with antineoplastic therapies. As with any ESA, for safety and economic considerations, biosimilar ESAs should be used according to the latest guidance and label.

In Europe there are two biosimilar ESAs marketed by different license holders: epoetin alfa (Abseamed^®^, Medice, Iserlohn, Germany; Binocrit^®^, Sandoz, Holzkirchen, Germany; Epoetin Alfa Hexal^®^, Hexal AG, Holzkirchen, Germany), and epoetin zeta (Retacrit^®^, Hospira, Lake Forest, IL, United States; Silapo, Stada Arzneimittel, Bad Vilbel, Germany). Retacrit^®^, for example, was one of the earliest biosimilar epoetins launched in Europe; post-marketing and clinical studies have demonstrated clinical efficacy and safety in oncology and nephrology indications, and exposure data reveal a growing population receiving this treatment (Michallet and Losem, 2016). In May 2018, Epoetin Hospira was licensed by the FDA for all indications of Epogen/Procrit, becoming the first and only biosimilar ESA to obtain approval in the United States. Now finally approved in the United States, biosimilar ESAs may, as in Europe, provide cost savings and increase patient access to other novel treatment approaches (Aapro et al., 2018b).

## Conclusion

**FIGURE 4:**
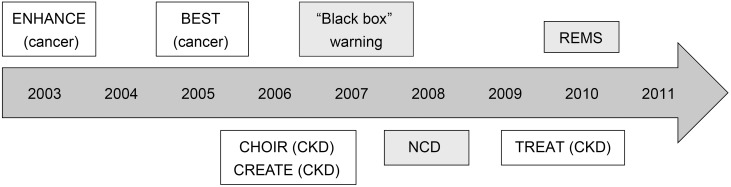
Timeline of key clinical trials and interventions between 2003 and 2011.

Erythropoiesis-stimulating agents have transformed the management of anemia in patients with CKD, cancer, and other indications, with some studies suggesting they improve QoL in certain subsets of patients with anemia (Evans et al., 1990; Parfrey et al., 2005; Drueke et al., 2006; Singh et al., 2006; Pfeffer et al., 2009). The results of key clinical studies have helped shape guidelines, but ESA use has been potentially impacted by regulatory actions such as the “black box” warning, REMS and the NCD (see Figure 4 for timeline). Although target Hb levels have been a key component of guidance, evolving data suggest that ESA dose and the speed at which Hb levels change in response to ESAs are also important considerations when treating anemic patients (US Food and Drug Administration, 2007a). Indeed, the latest product labeling no longer specifies a target Hb level, but use of the lowest ESA dose sufficient to reduce the need for transfusions. Biosimilar ESA products have been used successfully for many years outside the United States, with safety and efficacy comparable to originator products, bringing cost savings to patients and healthcare systems, and increased access to ESAs and other expensive drugs due to reallocation of resources. Further research will provide guidance on individualization of ESA therapy for different patients and indications so that the optimal benefit to risk ratio may be achieved.

**Table:** 

Trial	Disease (ESA)	Target Hb	Clinical outcomes
ENHANCE	Head and neck cancer (epoetin beta)	>14 g/dL (women); >15 g/dL (men)	Shortened PFS with ESA vs. placebo
BEST	Breast cancer (epoetin alfa)	12–14 g/dL	Lower 12-month OS with ESA vs. placebo
CHOIR	CKD (epoetin alfa)	13.5 g/dL vs. 11.3 g/dL	Cardiovascular endpoints and mortality increased in high-level Hb group
CREATE	CKD (epoetin beta)	Early therapy 13–15 g/dL vs. “salvage” 10.5–11.5 g/dL	Hastened progression to dialysis, more hypertensive episodes, improved QoL in 13–15 g/dL group
TREAT	CKD (darbepoetin alfa)	13 g/dL vs. placebo rescue (below 9 g/dL)	Higher incidence of stroke in 13 g/dL group. Cardiovascular events, death, and QoL similar for both groups

*BEST, Breast Cancer Erythropoietin Survival Trial; CHOIR, Correction of Hemoglobin and Outcomes in Renal Insufficiency study; CKD, chronic kidney disease; CREATE, Cardiovascular Risk Reduction by Early Anemia Treatment with Epoetin-beta study; ENHANCE, Erythropoietin in Head and Neck Cancer Trial; NCD, National Coverage Determination; REMS, Risk Evaluation and Mitigation Strategy; TREAT, Trial to Reduce Cardiovascular Events with Aranesp Therapy.*

## Author Contributions

MA, PG, KP, GR, SF, LA, and JW contributed to the conception, content and writing of this review article. All authors contributed to manuscript revisions, and read and approved the final version.

## Conflict of Interest Statement

MA is/was a consultant for Amgen, Hospira, Johnson & Johnson, Pfizer, Pierre Fabre, Roche, Sandoz, Teva Pharmaceutical Industries Ltd., and Vifor Pharma, and has received honoraria for lectures at symposia for Chugai Pharmaceutical Co., Ltd., Dr. Reddy’s, and Kyowa Hakko Kirin Co., Ltd. PG has presented at symposia for Johnson & Johnson, Amgen, Sandoz, and Pfizer. KP is a consultant for and has attended advisory boards for Pfizer Inc. GR is a consultant for Pfizer Inc. SF and LA are employees of Pfizer Inc. and hold stock in Pfizer. JW is a consultant for Pfizer Inc. and serves on the speakers’ bureau for Pfizer Inc.

## Abbreviations

AE: adverse event
ASCO: American Society of Clinical Oncology
ASH: American Society of Hematology
BEST: Breast Cancer Erythropoietin Survival Trial
CHOIR: Correction of Hemoglobin and Outcomes in Renal Insufficiency study
CIA: chemotherapy-induced anemia
CKD: chronic kidney disease
CREATE: Cardiovascular Risk Reduction by Early Anemia Treatment with Epoetin-beta study
EMA: European Medicines Agency
ENHANCE: Erythropoietin in Head and Neck Cancer (ENHANCE) trial
EORTC: European Organisation for Research and Treatment of Cancer
ERBP: European Renal Best Practice
ESA: erythropoiesis-stimulating agent
ESMO: European Society for Medical Oncology
EU: European Union
FDA: United States Food and Drug Administration
Hb: hemoglobin
KDIGO: Kidney Disease Improving Global Outcomes
NCCN: National Comprehensive Cancer Network
NCD: National Coverage Determination
NICE: National Institute for Health and Care Excellence
NKF-DOQI: National Kidney Foundation Dialysis Outcomes Quality Initiative
OS: overall survival
PFS: progression-free survival
RAE: reportable adverse event
QoL: quality of life
REMS: Risk Evaluation and Mitigation Strategy
TREAT: Trial to Reduce Cardiovascular Events with Aranesp Therapy
